# Analysis of long non-coding RNA expression profile of bovine monocyte-macrophage infected by *Mycobacterium avium* subsp. *paratuberculosis*

**DOI:** 10.1186/s12864-022-08997-5

**Published:** 2022-11-24

**Authors:** Yanhong Bao, Shuiyin Wu, Tianze Yang, Zi Wang, Yiming Wang, Xiuyun Jiang, Hongxia Ma

**Affiliations:** 1 College of Life Sciences, Jilin Agricultural University, Xincheng Street No.2888, Changchun, 130118 China; 2 College of Animal Science and Technology, Inner Mongolia University for Nationalities, Tongliao, 028000 China; 3 College of Animal Medicine, Jilin Agricultural University, Xincheng Street No. 2888, Changchun, 130118 China; 4 College of Life Sciences, Changchun Sci-Tech University, Changchun, 130600 P.R. China; 5 The Key Laboratory of New Veterinary Drug Research and Development of Jilin Province, Jilin Agricultural University, Xincheng Street No. 2888, Changchun, 130118 China; 6 The Engineering Research Center of Bioreactor and Drug Development, Ministry of Education, Jilin Agricultural University, Xincheng Street No. 2888, Changchun, 130118 China

**Keywords:** *M. avium* subsp. *paratuberculosis*, mRNA, LncRNA, High-throughput sequencing, Monocyte-macrophage

## Abstract

*Mycobacterium avium* subsp. *paratuberculosis* (MAP) is the causative agent of paratuberculosis. As a potential zoonotic pathogen, MAP also seriously threatens human health and social security. At present, long non-coding RNA (lncRNA) has attracted wide attention as an useful biomarker in various diseases. Therefore, our study analyzed the lncRNA expression profiles and lncRNA-mRNA regulatory network of MAP infected bovine monocytes-macrophages and uninfected bovine cells by high-throughput sequencing. A total of 4641 differentially expressed lncRNAs genes were identified, including 3111 up-regulated genes and 1530 down-regulated genes. In addition, lncRNA-mRNA interaction analysis was performed to predict the target genes of lncRNA. Among them, after MAP infection, 86 lncRNAs targeted to mRNA, of which only 6 genes were significantly different. The results of Gene Ontology (GO) enrichment analysis showed that the differentially expressed genes significantly enriched in functional groups were related to immune regulation. Multiple signal pathways including NF-κB, NOD-like receptor, Cytokine-cytokine receptor, Toll-like receptor signaling pathway, Chemokine signaling pathway, and other important biochemical, metabolic and signal transduction pathways were enriched in Kyoto Encyclopedia of Genes and Genomes (KEGG). In this study, analysis of macrophage transcriptomes in response to MAP infection is expected to provide key information to deeply understand role of the pathogen in initiating an inappropriate and persistent infection in susceptible hosts and molecular mechanisms that might underlie the early phases of paratuberculosis.

**Supplementary Information:**

The online version contains supplementary material available at 10.1186/s12864-022-08997-5.

## Introduction

Paratuberculosis, also known as Johne’s disease (JD), can cause chronic enteritis in cattle and other ruminants. This disease is endemic in many ruminant herds worldwide [1] and has caused significant economic losses to the dairy industry. In a survey of 48 countries, paratuberculosis was found to be very common in livestock. In about half of the countries, more than 20% herds are infected with paratuberculosis [2]. Paratuberculosis has been found of more than 68% of cattle herds in the United States [3]. In Canada, the economic losses associated with JD in dairy herds were estimated to be more than US$35–57/year/cow [4]. The disease may cause profuse chronic diarrhea, submandibular edema, weight loss despite normal appetite, malnutrition, lethargy, and even death [5]. *Mycobacterium avium* subsp. *paratuberculosis* (MAP), pathogen of paratuberculosis, is a slowly growing fastidious acid-fast bacterium belonging to the *Mycobacterium avium* complex (MAC). MAP can infect a variety of wild ruminants, as well as common mammals including cattle and sheep, where cattle are the most common host for MAP infection [6–8]. The most common symptoms of this disease in cattle are loss of milk production, weight loss and diarrhoea, whereas in sheep and goats, the symptoms are emaciation, anorexia and severe disability [9]. In addition to cattle, sheep and goats, MAP has been found in ruminants such as white-tailed deer [10], red deer [11], elk [12] and non-ruminants such as rabbits and foxes [13], and even primates such as mandrills and macaques [14], indicating the wide adaptability of this *mycobacterium* to host species. This extensive host species may lead to the wide spread of MAP, which makes it difficult to control JD on a global scale. Due to the widespread and impact of JD in global livestock and the huge economic losses it cause, it has been recognized by the World Organization for Animal Health as one of the most important infectious diseases. In addition, MAP may be associated with the development of human Crohn’s disease, type I diabetes, multiple sclerosis, or rheumatoid arthritis [15–18].

The main reasons why MAP is prevalent and difficult to eradicate are the amazing speed of transmission and the widespread and susceptibility of the host. The pathogen can be transmitted by fecal–oral route, feces and milk of subclinical or clinically infected animals, allowing MAP to spread to susceptible calves, the environment, and dairy products for human consumption [19, 20]. Milk containing MAP is particularly worrying because there is increasing evidence that MAP is a zoonotic pathogen [21]. MAP is an obligate intracellular pathogen that can multiply in host’s macrophages. A mouse model showed that the junction between MAP and intestinal mucosa and the translocation of MAP through intestinal mucosa were mediated by M cells and intestinal cells after MAP was ingested [22]. In the submucosa, MAP was uptaken by macrophages that play a critical role in the host–pathogen interaction of JD. They can not only mediate the destructive effect of MAP, but when macrophages are attacked and subverted by MAP, they can be transformed into safe havens for pathogens to survive, proliferate and spread [23]. In different periods of infection, MAP promotes or inhibits apoptosis respectively [24].

Long non-coding RNA (lncRNA) is a type of non-coding RNA with a length greater than 200 nt, which is mainly transcribed from the antisense strand and spacer region of protein-coding genes. LncRNA has been shown to participate in many important regulatory processes such as X chromosome silencing, genome imprinting, chromatin modification, transcription activation, transcription interference, and nuclear transport [25]. Besides, as a competitive endogenous RNA (ceRNA), lncRNA can affect the pathophysiological environment of different organisms and ultimately affect gene expression [26]. The lncRNA-mediated ceRNA network affects the ischemic stroke (IS) process, especially the immune response after stroke [27]. LncRNA has been reported to be involved in a variety of diseases and is considered as molecular biomarkers for disease diagnosis and treatment [28–31]. LncRNAs are also regarded as critical regulators of gene expression at both the transcriptional and post-transcriptional levels. The lncRNA located in nucleus participates in gene regulation at epigenetic and transcription level, for example, DNA methylation [32]. At the same time, lncRNA located in the cytoplasm participates in gene regulation at post-transcriptional and translational levels, such as regulation of mRNA metabolism [33]. The expression level of lncRNA is usually lower than that of mRNA, but lncRNA has stronger tissue specificity [34], indicating an indispensable role in the process of cell type specificity [35]. So far, there are few studies on the lncRNA expression profile of MAP-infected bovine peripheral blood mononuclear macrophages. Therefore, compared with studies in human diseases and mouse disease models, there are still many unsolved issues of lncRNA’s immune regulation and genetic changes in response to bacteria and other external infections in cattle.

The potential zoonotic threat of MAP and the huge economic losses caused by MAP prompt the formulation of effective management strategies to prevent the spread of the disease. Therefore, understanding the pathogenic mechanism of MAP and its transmission mode is crucial for vaccine development and better control of the disease progression. It is well known that macrophages are the target cells where MAP is able to survive and multiply and play a crucial role in the host–pathogen interaction. Currently, macrophages have been used as pathogenic models to study the pro- or anti-inflammatory transition of immune cell effector function following MAP exposure due to its crucial role in the development and control of MAP disease. To better study the pathogenic mechanism and immune methods of MAP in cattle, high-throughput sequencing was performed to analyze the lncRNA expression profile and lncRNA-mRNA interaction network of MAP infected bovine monocyte-derived macrophages (MDM).

## Results

### Cell total RNA extraction results

The total RNA concentration and quality test results are shown in Supplementary Table 1 and Supplementary Fig. 1. The RNA quality is good and meets the requirements for continuing to construct a library and sequencing.

### Analysis of the overall expression level of lncRNA

**Fig. 1:**
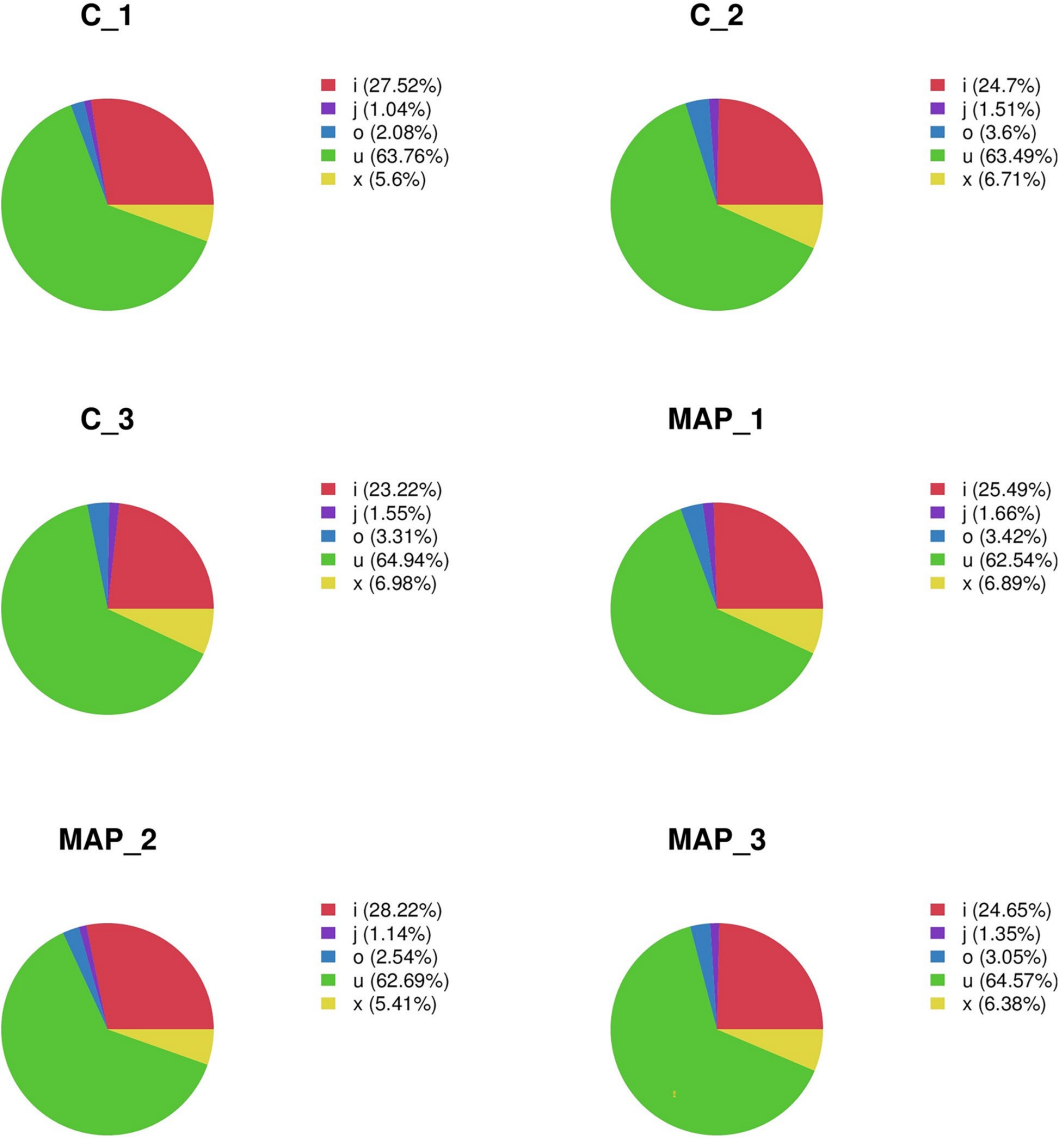
Pie chart of the proportion of lncRNA with different class codes in each sample

**Fig. 2:**
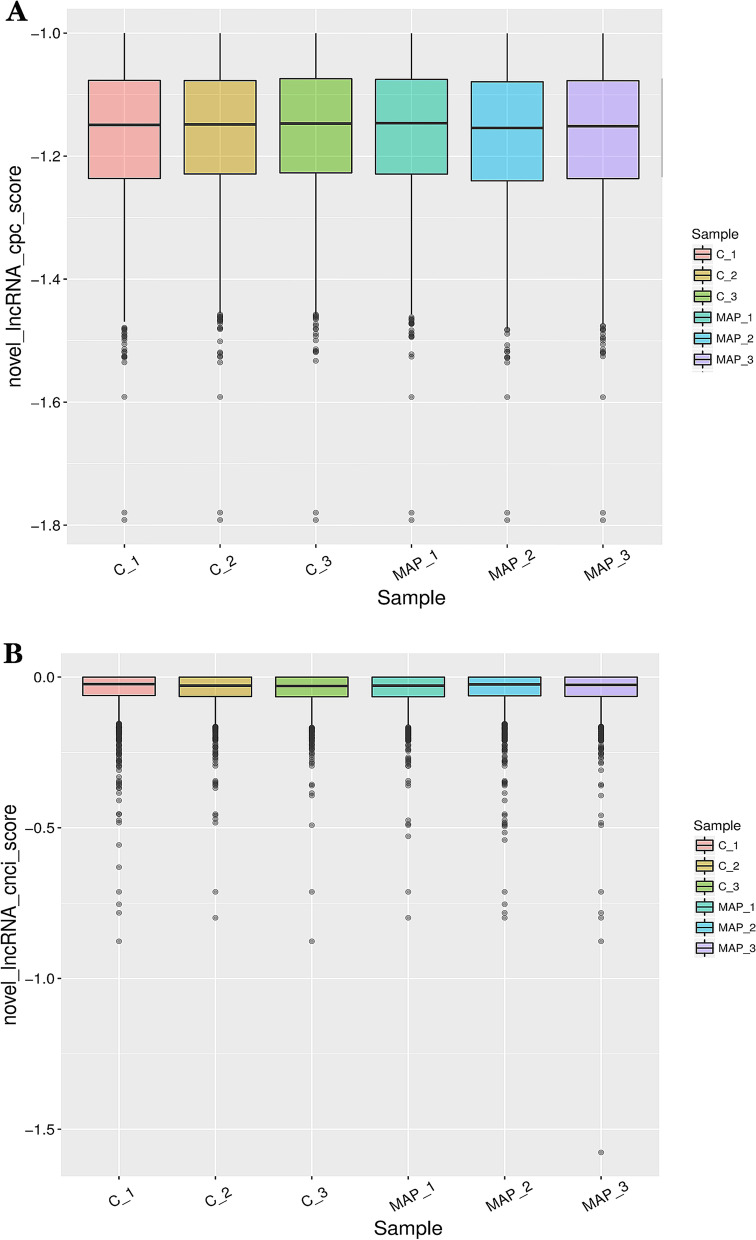
**A** Statistical boxplots of scores for lncRNAs in each sample predicted using CPC. **B** Statistical boxplots of scores for lncRNAs in each sample predicted using CNCI

The expression level of lncRNA mainly uses FPKM (Fragments Per Kilobase of exon model per Million mapped reads) to measure the abundance value of gene expression, and the expression abundance of known genes in different samples is counted by FPKM. FPKM represents the number of sequenced fragments contained in every thousand transcript sequenced bases per million sequenced bases. Simply put, the FPKM value can be understood as the expression level of lncRNA. The five types of class code definition information are shown in Supplementary Table 2. All lncRNA expression profile information is shown in Supplementary Table 3. We made statistics on the percentage of lncRNA different class code in each sample, and the pie chart is shown in Fig. 1. The score statistical box diagram of lncRNA CPC (Coding Potential Calculator) and CNCI (Coding-Non-Coding Index) in each sample is shown in Fig. 2A and B. Among the 4928 novel non-coding transcripts predicted by CPC and CNCI, most novel lncRNAs were defined on chromosomes 3 and 10, with 241 and 285 lncRNAs, respectively.

### Visualization results of lncRNA genome in different samples

**Fig. 3:**
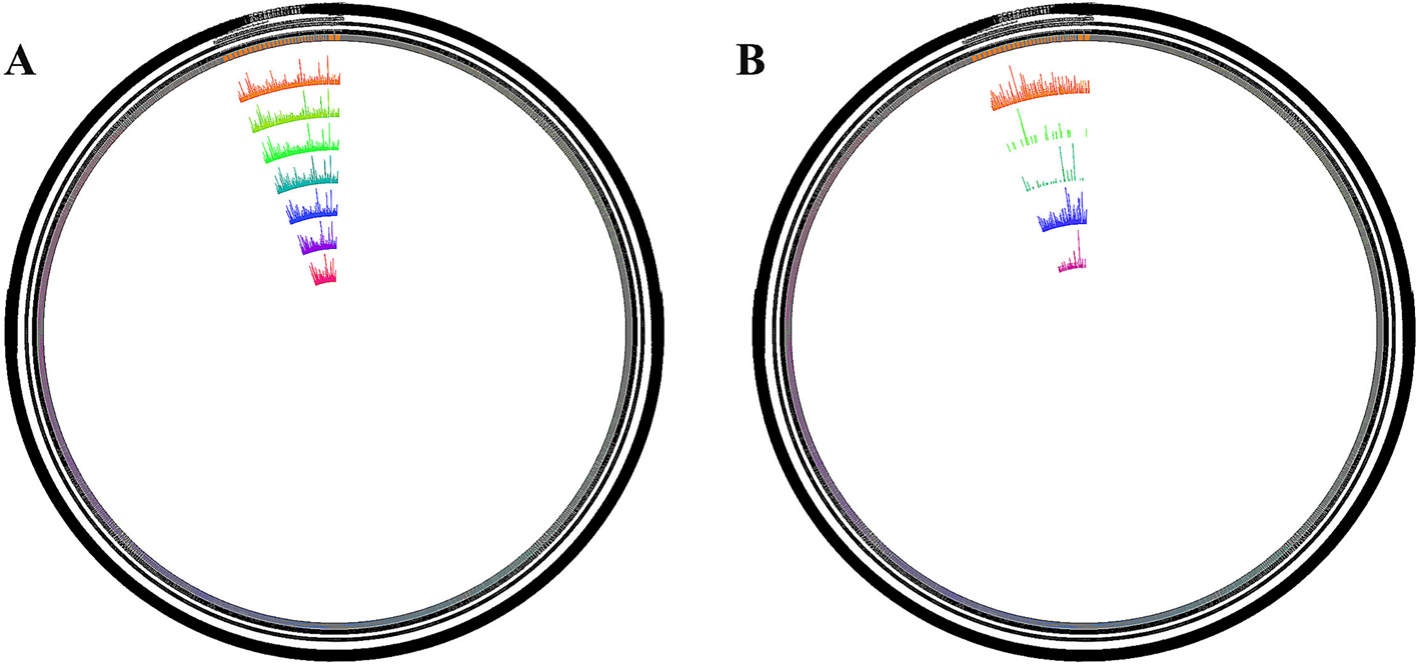
**A** Genome mapping according to different classifications of lncRNA. **B** Genome mapping according to lncRNA in different samples

In order to show the distribution of lncRNA candidates in the chromosomes more intentionally, we used circos software to perform genome mapping on the lncRNAs obtained from the screening. It is mainly divided into two parts for display, one is for genome mapping according to different classifications of lncRNA, and the other is for genome mapping according to lncRNA in different samples. When mapping, every chromosome is based on every 25 mb. When mapping lncRNA genome visualization in different samples, the expression quantity of lncRNA in each segment is counted and plotted, as shown in Fig. 3A. The number of lncRNAs in each segment should be counted and plotted when analyzing the genome visualization of different types of lncRNAs, as shown in Fig. 3B.

### Analysis of differential lncRNA expression results

**Fig. 4:**
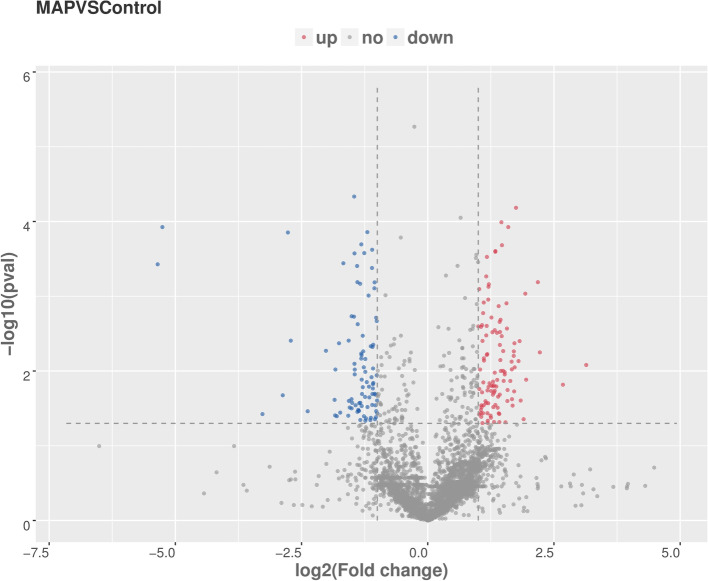
Volcanic map analysis of differential gene expression level. A scatter plot shows the correlation of gene abundance. The red dot, blue dot and gray dot is signify up-regulation, down-regulation and not different respectively

**Fig. 5:**
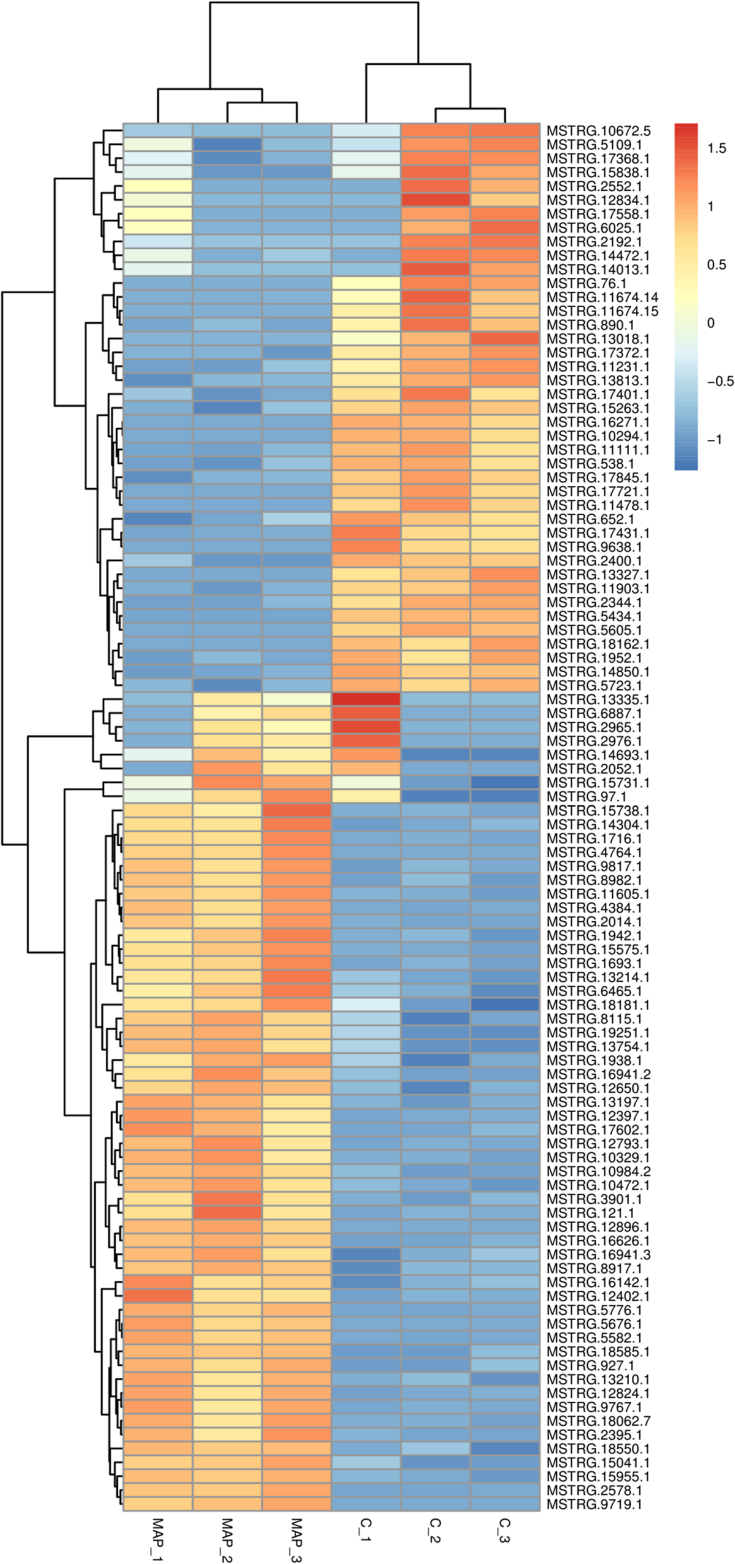
Cluster analysis of differential gene expression level. The abscissa is the sample and the ordinate is the gene. Red indicates high expression gene, and dark blue indicates low expression gene

The experimental results showed that there were a total of 4641 differentially expressed lncRNA genes (Supplementary Table 4), including 3111 up-regulated genes and 1530 down-regulated genes. There were 102 genes with significant differences among the up-regulated genes, and 91 genes with significant differences among the down-regulated genes. In order to show the overall distribution of differentially expressed genes more intuitively, volcanic maps (Fig. 4) and heat maps (Fig. 5) were drawn for all genes in differential expression analysis.

### Comparison of lncRNA and mRNA structural characteristics

**Fig. 6:**
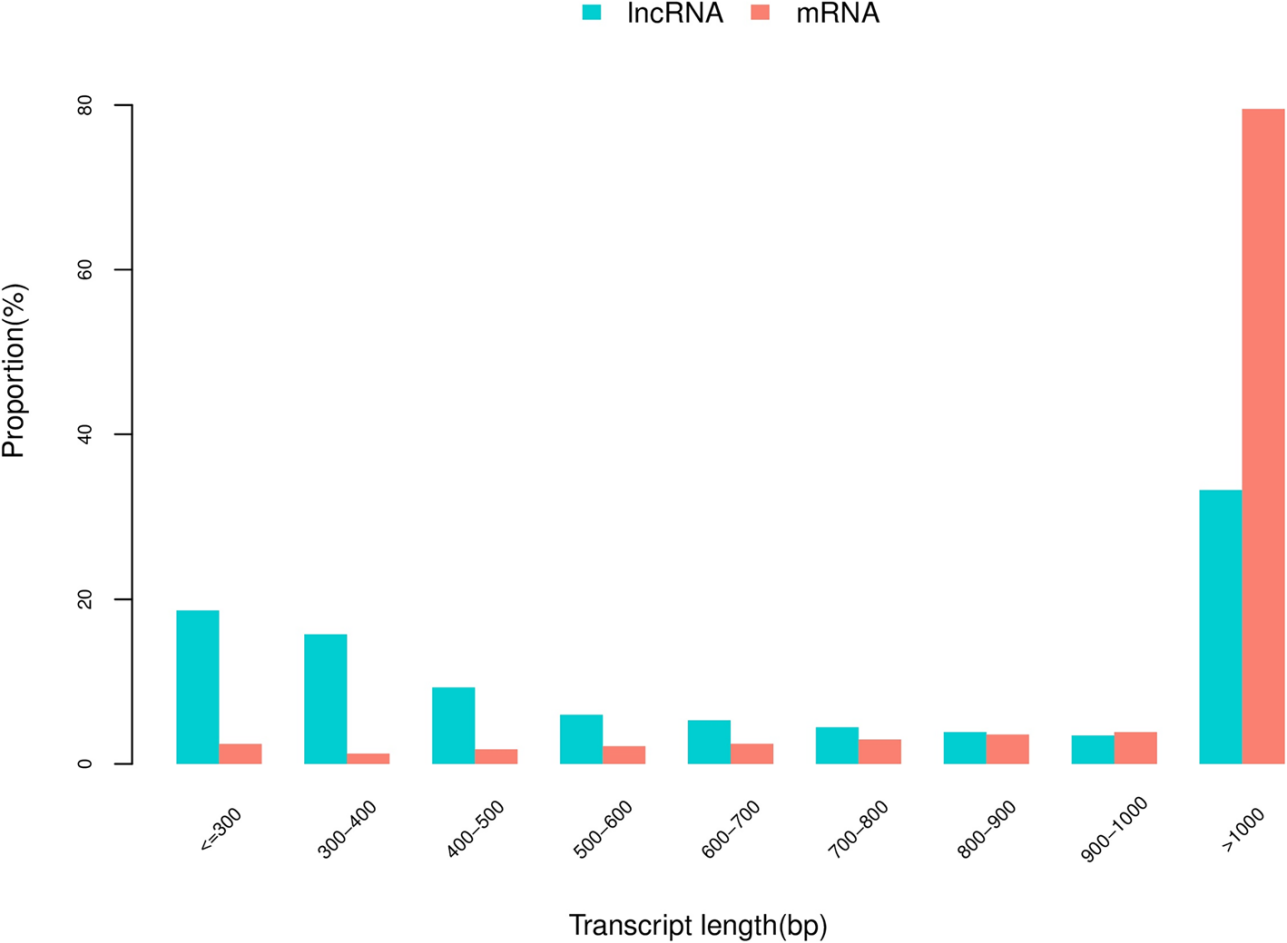
Statistics and comparison of lncRNA and mRNA length

**Fig. 7:**
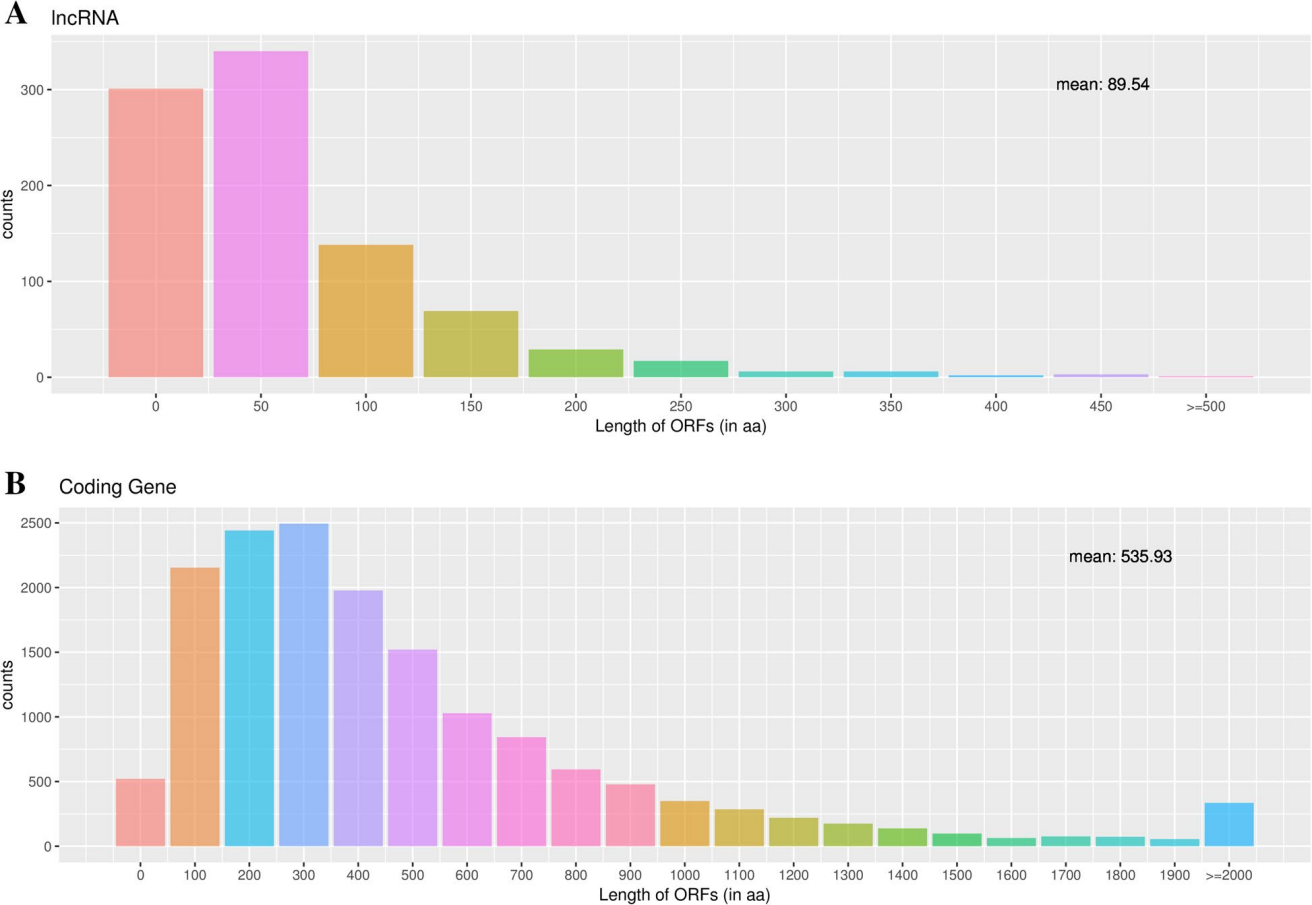
**A** The ORF length hierarchy of translation in lncRNA. **B** The ORF length hierarchy of translation in mRNA

**Fig. 8:**
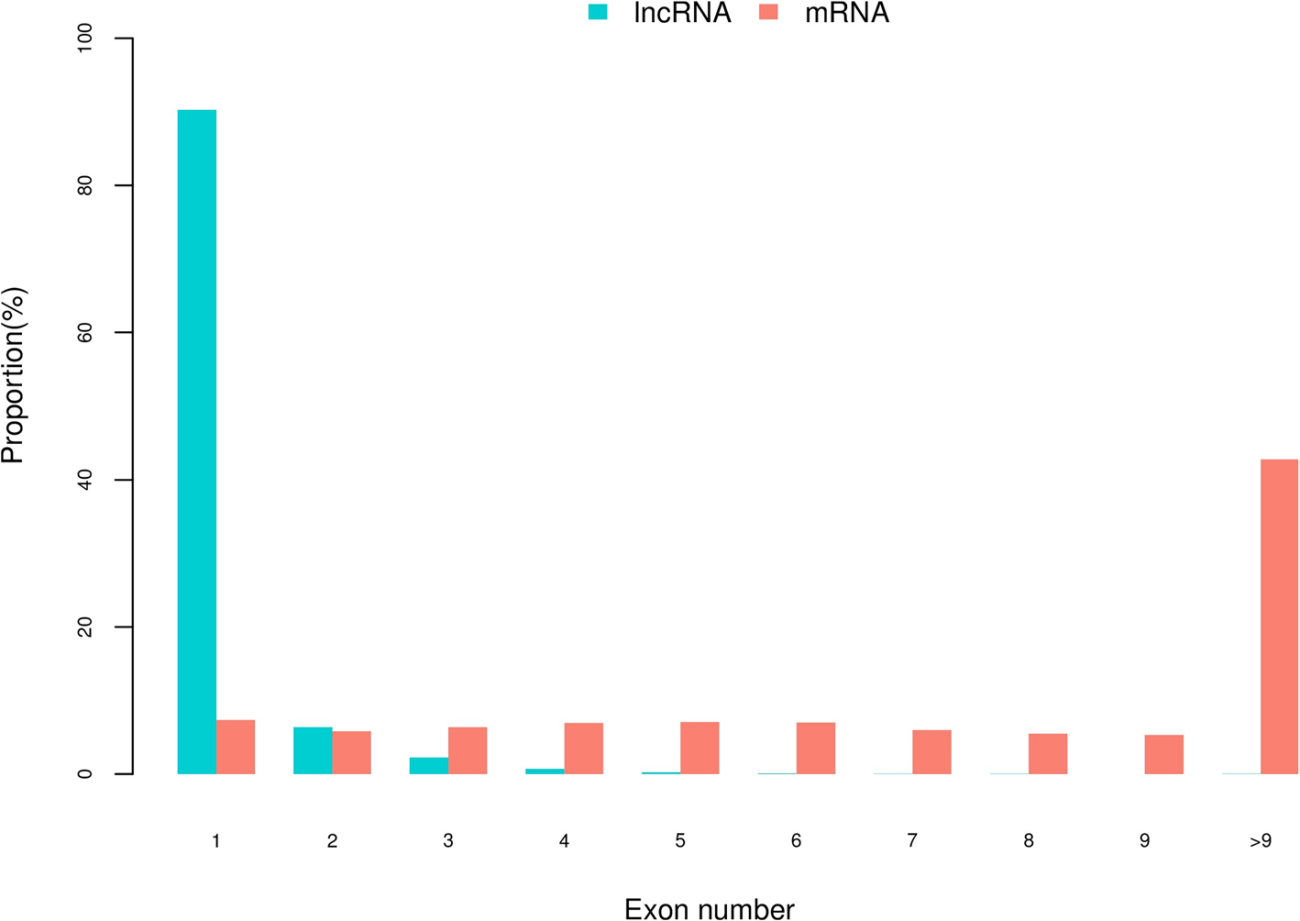
Statistics of the number of lncRNA and mRNA exons

**Fig. 9:**
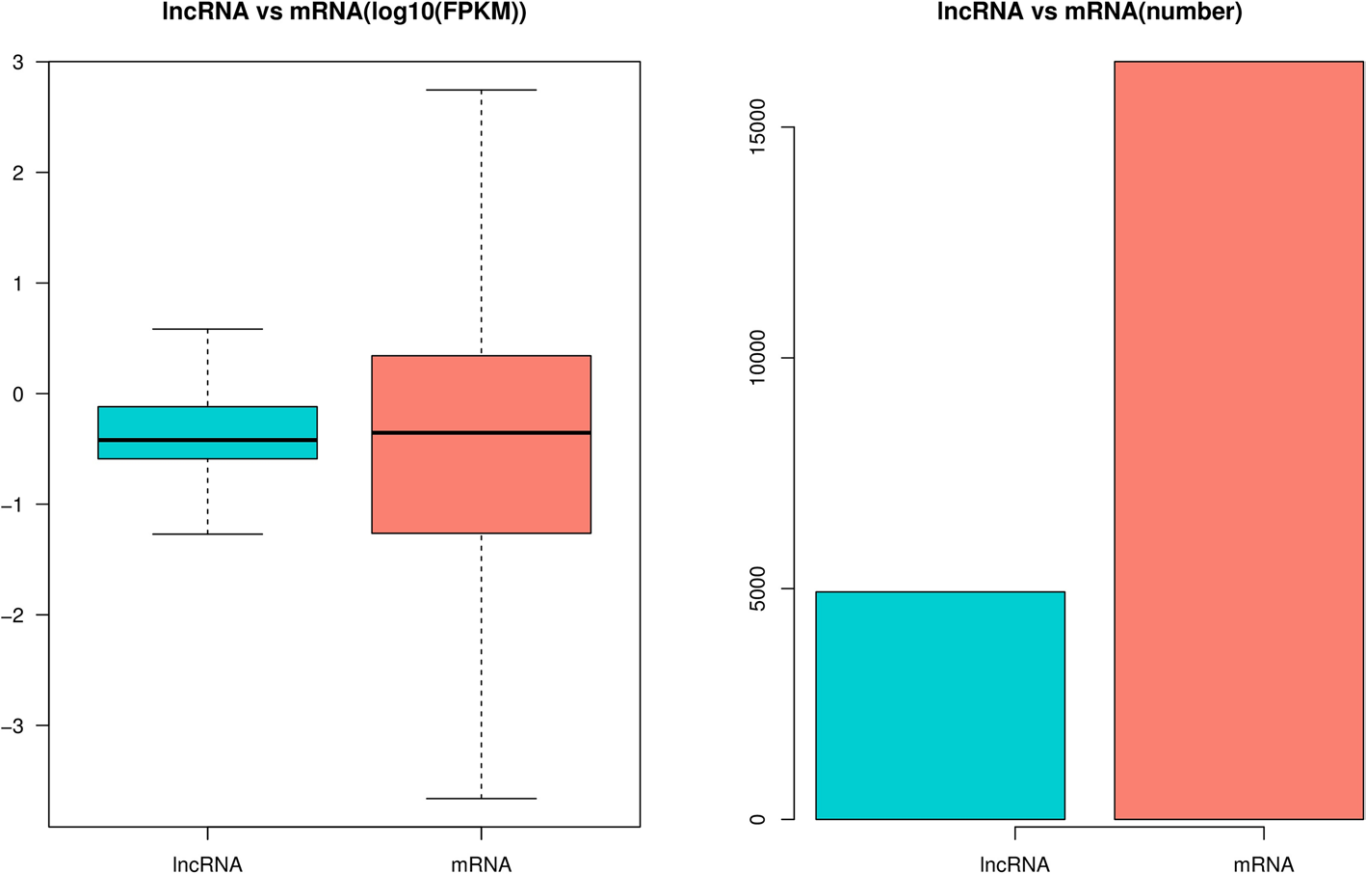
Statistics of lncRNA and mRNA expression levels

LncRNA is also called long-chain non-coding RNA, with a length greater than 200 nt, which is mainly transcribed from the antisense strand and spacer region of protein-coding genes. They regulate the expression level of genes in the form of RNA at multiple levels (epigenetic regulation, transcription regulation, post-transcriptional regulation, etc.). The biological content is quite abundant, accounting for about 4–8% of RNA (mRNA accounts for about 1–2%). Therefore, the follow-up functional annotation of lncRNA can be made by analyzing the length and expression correlation of lncRNA and mRNA (Supplementary Table 5 and Fig. 6). It has been reported that lncRNA has no obvious ORF and has very low protein coding potential [36, 37]. We compare the length of the ORF of lncRNA and mRNA translation and discuss the differences between the two (Supplementary Tables 6 and 7). The principle of ORF analysis is mainly based on the six-frame translation principle of nucleic acid (Fig. 7A and B). In addition, the number of lncRNA and mRNA exons (Supplementary Table 8 and Fig. 8) and the expression level of lncRNA and mRNA (Supplementary Table 9 and Fig. 9) were also counted.

### lncRNA and mRNA interaction analysis

#### lncRNA target gene prediction

**Fig. 10:**
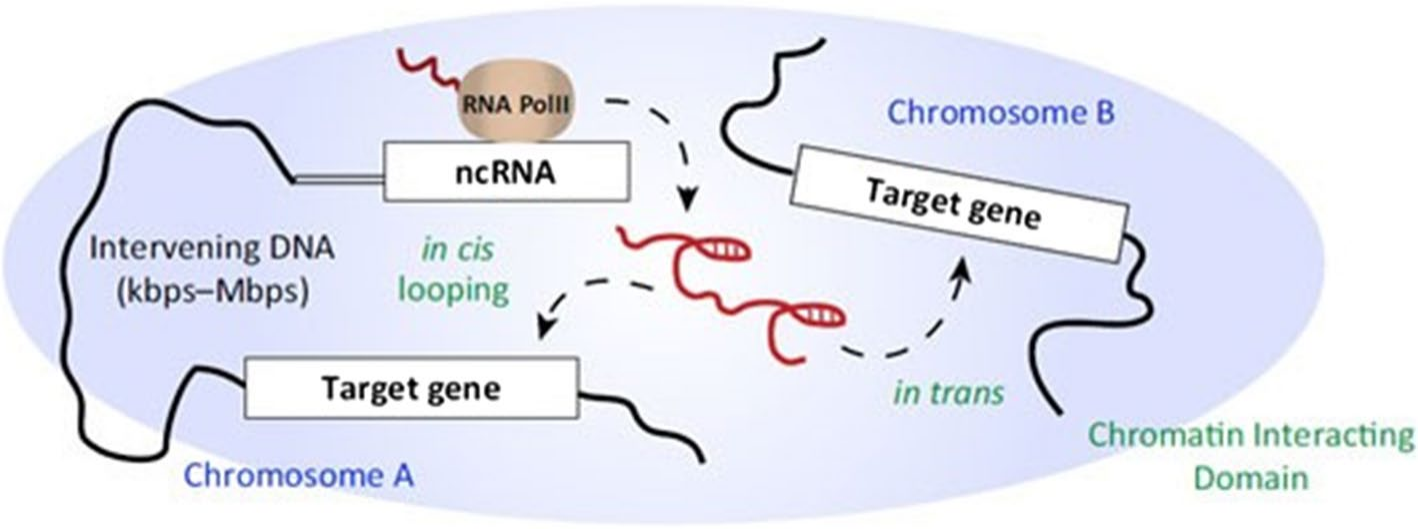
Schematic diagram of lncRNA regulation mechanism

There are two main types of lncRNA regulation. One is cis-regulation, that is, lncRNA regulates the expression of its neighboring genes. The cis-regulation of target genes by lncRNA is mainly based on the prediction of the positional relationship, which defines the differentially expressed lncRNA and differentially expressed mRNA in the upper and lower reaches of the chromosome in the range of 100 kbp, and this type of lncRNA constitutes cis-regulation [38, 39]. Specifically, cis-regulation mainly relies on cis-acting elements. Cis-acting elements refer to sequences that can affect gene expression in the flanking sequences of genes, including promoters, enhancers, regulatory sequences and inducible elements, etc. The role is to participate in the regulation of gene expression in the nucleus, and cis-acting elements are usually transcribed into non-coding RNA; the second type of regulation is trans regulation, that is, lncRNA regulates the expression of genes across chromosomes. The trans regulation of target genes is mainly based on the amount of free energy required to form a secondary structure between lncRNA and mRNA sequences. If the binding of two sequences requires very low free energy, there may be an interaction between them. In this article, we mainly conduct predictive analysis of differential mRNA and differential lncRNA regulated by cis. The schematic diagram of lncRNA regulation mechanism is shown in Fig. 10.

In this paper, only the upstream and downstream 100 kbp mRNA and lncRNA are predicted in cis-regulation. A total of 86 lncRNAs were targeted to mRNA in MAP-infected and control MDM cells (Supplementary Table 10). Among them, only 6 targets have significant differences, see Table 1. Among them, the differential lncRNAs are located on the 2nd, 5th, 6th, 11th, 14th and 19th chromosomes, respectively, and the corresponding target mRNAs are CYP1B1, IMPA1, FXR2, ECE1, BHLHE41 and IL-8.

**Table 1:** LncRNA targets differential genes

mRNA			lncRNA				
gene name	chr	Description	gene id	chr	class code	cis/trans	cis location
CYP1B1	chr11	cytochrome P450 family 1 subfamily B member 1	MSTRG.2049	chr11	u	cis	10 K
IMPA1	chr14	inositol monophosphatase 1	MSTRG.4384	chr14	x	cis	100 K
FXR2	chr19	fragile X mental retardation syndrome-related protein 2	MSTRG.7505	chr19	u	cis	10 K
ECE1	chr2	endothelin converting enzyme 1	MSTRG.8917	chr2	u	cis	100 K
BHLHE41	chr5	basic helix-loop-helix family member e41	MSTRG.15575	chr5	u	cis	100 K
IL-8	chr6	Interleukin-8	MSTRG.16271	chr6	u	cis	100 K

#### Differential gene GO and KEGG enrichment analysis

After infecting bovine monocyte-macrophage cells with MAP, in order to understand the specific functional roles of the differentially expressed genes, GO and KEGG enrichment analysis were performed on the differentially expressed genes.

**Fig. 11:**
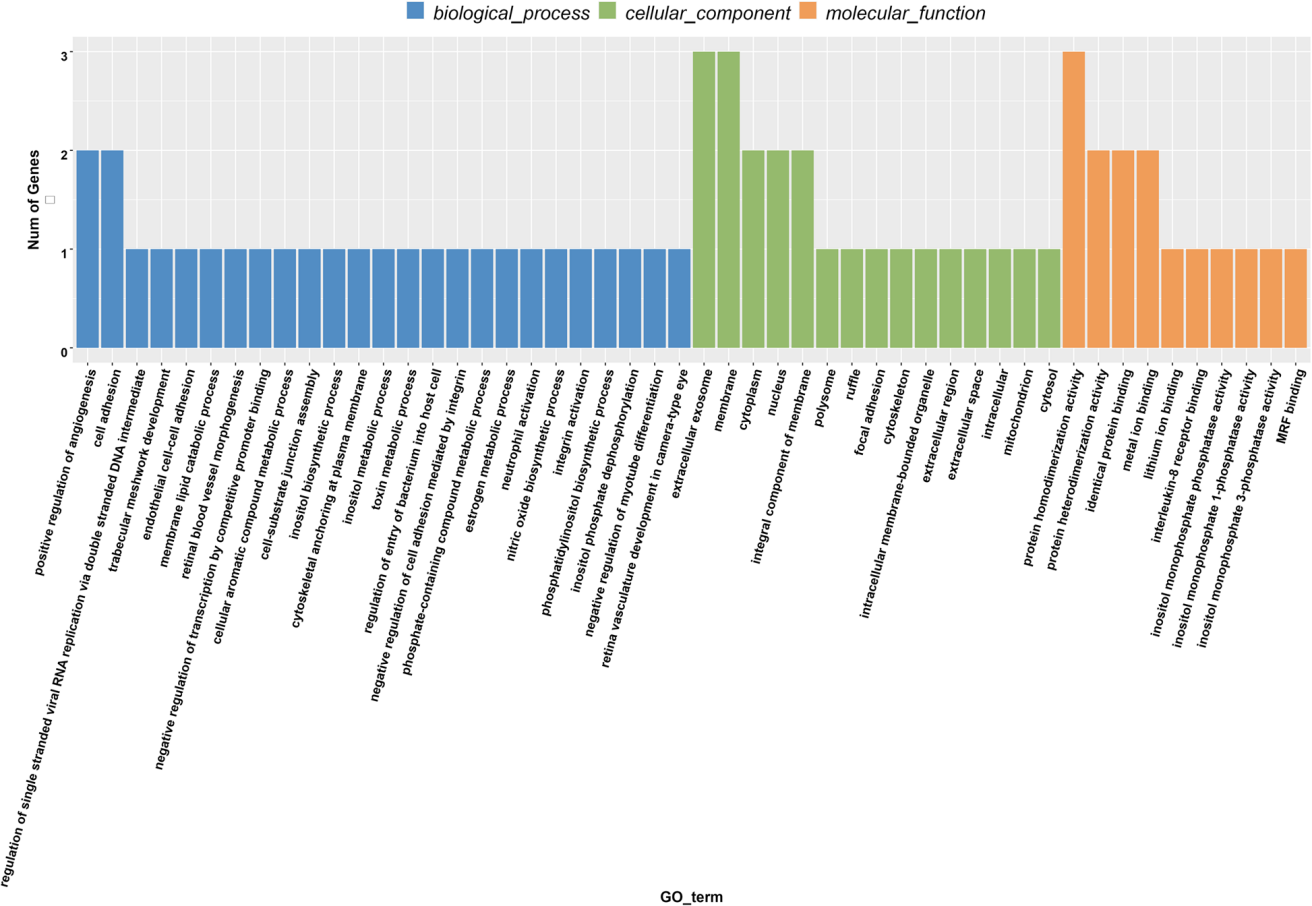
Histogram of GO enrichment of differential genes

GO has a total of three ontology, which describe the molecular function, cellular component, and biological process of the gene. The statistical description of these three ontologies in the text is shown in Supplementary Table 11. Among all the selected lncRNA differential genes, we divided the GO annotations corresponding to these differential genes into three categories according to Biological Process, Cellar Component and Molecular Function, and classified the GO functions in each category according to the target genes annotated. The numbers are sorted from high to low, and the top 25, 15, and 10 GO categories are selected for mapping (Fig. 11). In the classification of Biological Process, the most significant group was the regulation of single stranded viral RNA replication via double stranded DNA intermediate (GO: 0,045,091) GO functional group, followed by the trabecular meshwork development (GO: 0,002,930) GO functional group, with respectively 1 annotated gene IL-8 and 1 annotated gene CYP1B1. In the Molecular Function classification, the most important GO functional groups are the lithium ion binding functional group (GO: 0,031,403) with 1 annotated gene IMPA1 and the interleukin-8 receptor binding (GO: 0,005,153) functional group with 1 annotated gene IL-8. In the classification of Cellar Component, the most prominent functional group was the polysome (GO: 0,005,844) group with 1 annotated gene FXR2, followed by the ruffle (GO: 0,001,726) function group with 1 annotated gene TLN1.

**Fig. 12:**
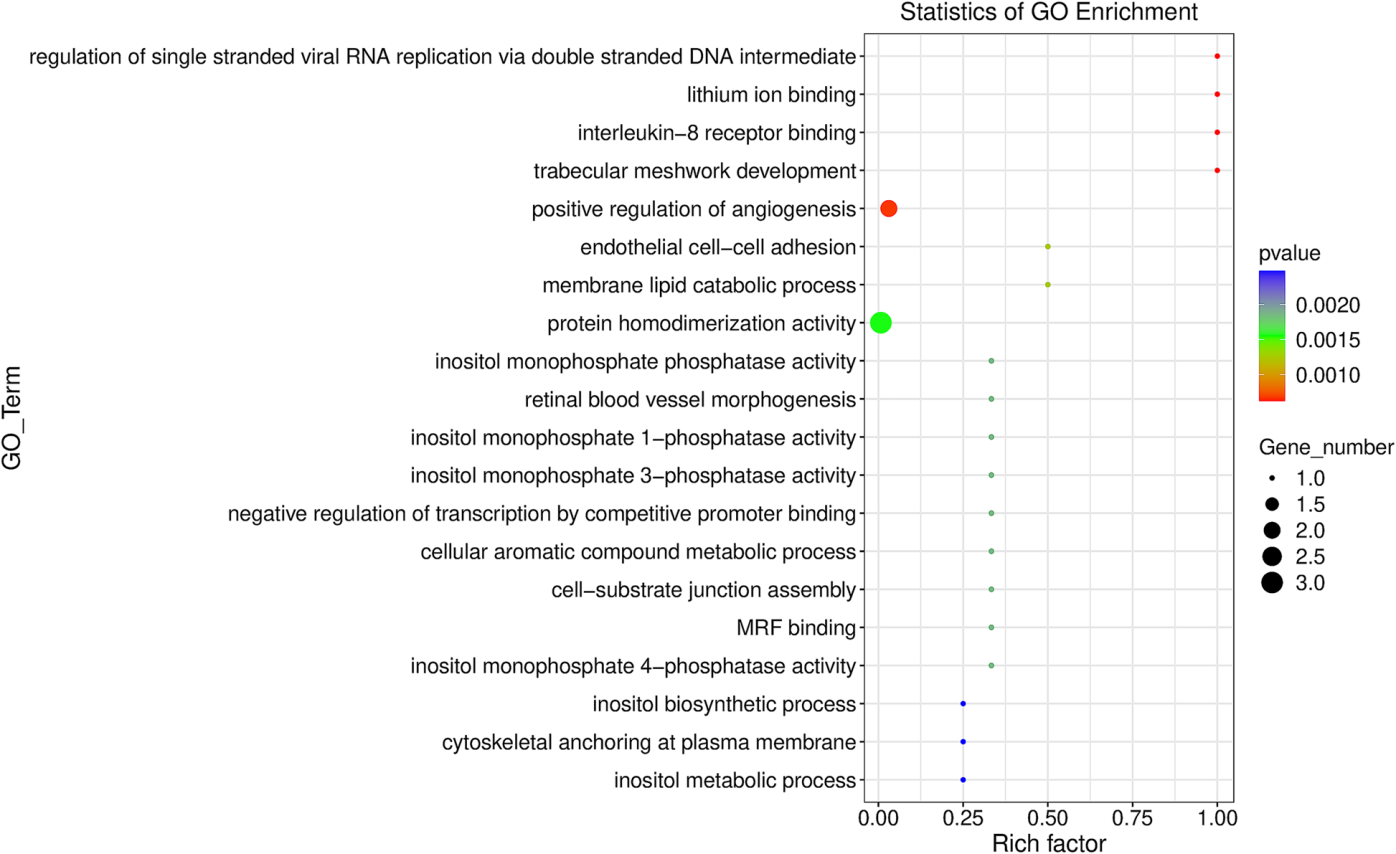
The scatter plot of GO enrichment analysis. The abscissa Rich factor indicates the number of differential genes located in the GO/the total number of genes located in the GO. The larger the Rich factor, the higher the enrichment degree of GO; The ordinate is GO Term, which is the GO function comment. In the scatter plot, the size of the dot represents the number of genes with significant difference that S gene number matches to a single GO, and the color of the dot represents the *P* value of enrichment analysis (i.e., the significance of enrichment)

The basic unit of GO is term, and each term corresponds to an attribute. The GO functional significance enrichment analysis first maps all the significantly differentially expressed genes to each term of the Gene Ontology database, calculates the number of genes in each term, and then applies the hypergeometric test to find out the significant difference compared with the whole genome background. Significantly enriched GO entries among differentially expressed genes. In this experiment, ggplot2 was used for GO enrichment analysis and Top20 GO_term was plotted, and the results were displayed in a scatter plot (Fig. 12).

**Fig. 13:**
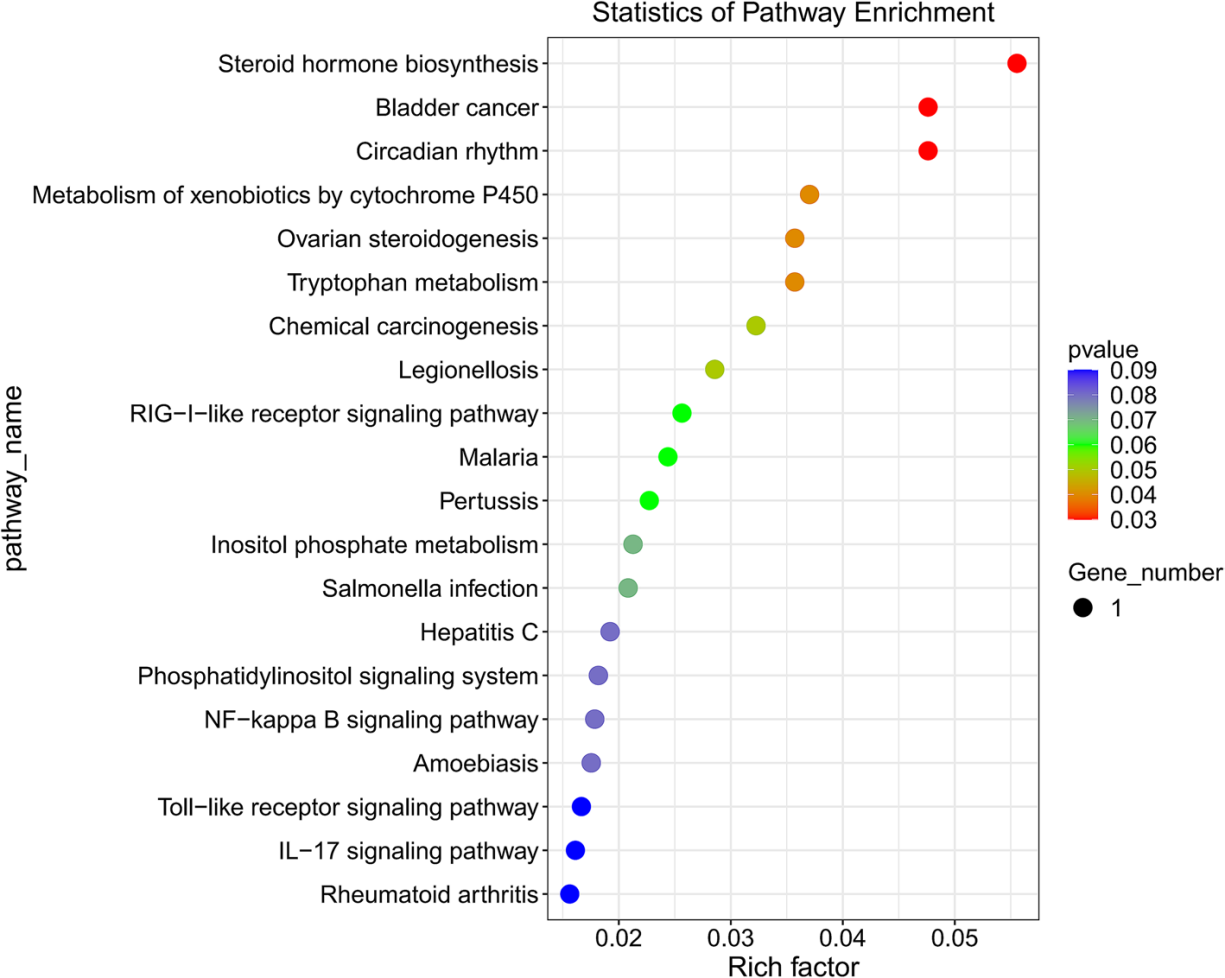
Scatter plot of KEGG enrichment of differential genes. The abscissa Rich factor indicates the number of differential genes located in the KEGG/the total number of genes located in the KEGG. The ordinate is Pathway term, that is, KEGG metabolic pathway [40–42]

In organisms, different genes coordinately perform their biological functions. Pathway-based analysis helps to further understand the biological functions of genes. KEGG is the main public pathway database. The results of KEGG enrichment analysis in this study are shown in Supplementary Table 12. Use ggplot2 to perform enrichment analysis on KEGG, and select the top 20 pathways according to the enriched *P* value to display the results in a scatter plot (Fig. 13). The KEGG pathway map shows that the enriched target genes are involved in multiple important biochemical, metabolic and signal transduction pathways, such as NOD-like receptor signaling pathway (ko04621), NF-κB signaling pathway (ko04064), Toll-like receptor signaling pathway (ko04620), Chemokine signaling pathway (ko04062), and Cytokine-cytokine receptor interaction (ko04060).

## Discussion

MAP can cause paratuberculosis in a variety of domestic and wild animals, which has resulted in serious economic losses and threats to human health and public health security. The disease is chronic, consumptive, and incurable. MAP is mainly transmitted through the fecal–oral route. Besides, it can also be spread via saliva, uterine fluid or semen [43, 44]. Another study has shown that MAP can also be transmitted through aerosols [45]. There is also a hazard of lateral transmission between herds.

Compared with the research progress of lncRNA in other species such as humans and mice, there are few studies on the mechanism of lncRNA regulating bovine immune response. Recently, with the application of high-throughput sequencing technology, studies on the identification of lncRNA expression in cattle immune-related tissues, cells and differences in expression during the period of bacterial/virus infection are gradually increasing. High-throughput sequencing technology has greatly enhanced the ability to understand interaction between host macrophages and *mycobacterium* pathogens. Therefore, high-throughput sequencing was used in this study to analyze the transcriptome of MAP-infected bovine mononuclear-macrophages. As mentioned earlier, most of the transcriptional changes in MDM induced by MAP infection occurred within first 6 h [46]. Therefore, in our study, the lncRNA expression profile and lncRNA-mRNA expression network of MDMs 6 h after infection were analyzed. In 2019, RNA-Seq technology was applied to study the changes of mRNA and lncRNA genome expression profile of MAP infected bovine macrophages in vitro by Pooja Gupta et al. [47]. The results showed that differentially expressed lncRNA was highly positively correlated with adjacent differential protein coding genes, and some differentially expressed lncRNA adjacent protein coding genes were related to immune response related pathways. This study is the first time to put forward a new view on the role of lncRNAs and its interaction with mRNAs in the process of cattle MAP infection. In 2021, Andrew Marete et al. [48] studied the potential role of lncRNA in mononuclear macrophages of dairy cows naturally infected with MAP. The results showed that the lncRNA target genes were significantly enriched in biological process involved in immunity and nucleic acid regulation. The study demonstrates the potential role of lncRNA in host immunity and the potential candidate genes and pathways that may play in response to MAP infection. Coincidentally, our results showed that differentially expressed target genes of lncRNA are also related to inflammation and immune response. Therefore, lncRNA plays an important role in MAP infected macrophages. By analyzing the reasons, the complex numbers of infection of MAP-infected cells in these two papers were 100:1 and 10:1, respectively, while the complex numbers of MAP infection in this paper were 5:1. Therefore, it is speculated that this may be caused by the difference in the complex numbers of infection. Additionally, in this study, the proportion of new lncRNA found on chromosomes 1, 2, 7 and X exceeds 60% of the total lncRNA. X chromosome accounts for the highest proportion, and the newly found lncRNA on chromosome 18 is 54.8%, which are both consistent with the research results of Andrew Marete et al. [48].

Presently, more and more evidences showed that lncRNA widely attended host’s response to pathogen invasion. Early studies have shown that the presence of several lncRNAs in cattle has an immunomodulatory effect on host’s immune response to *Staphylococcus aureus* infection. In a cell model of bovine *S. aureus* mastitis, lncRNA-TUB and H19 mediated *Escherichia coli*-induced inflammatory factor secretion and *S. aureus* adhesion to epithelial cells [49, 50]. Gupta et al. [51] found that in bovine paratuberculosis induced by *Mycobacterium paratuberculosis*, lncRNA (XLOC-033995) can regulate the expression level of its adjacent inflammatory signal factor TNFAIP3 and participate in immune response related to NF-κB, organelle fission pathways, which affects the inflammatory response process of macrophages to infection, and ultimately regulates the pathogenesis of bovine paratuberculosis. During breeding process, bovine viral diarrhea virus (BVDV) infection can severely affect digestive system and lead to persistent diarrhea and enteritis of a cow. Due to BVDV replicates in the infected Madin-darby bovine kidney cells (MDBK), more and more genes are activated and participate in the immune response, where the number of lncRNAs involved in regulation also increases significantly [52]. Moreover, lncRNA is also related to other diseases, such as liver metastasis, Alzheimer’s disease and colorectal cancer [53–55].

In this study, MAP was used to infect bovine monocyte-macrophages for 6 h. The most obvious expression increase in monocyte-macrophages is IL-8, a chemokine that in charge of maintaining balance between physiological reactions and pathological processes in many body systems. Monocytes and macrophages represent principal cellular sources of IL-8 [56]. IL-8 is a strong neutrophil chemotactic agent, which can enhance the killing effect of neutrophils and macrophages on mycobacteria [57]. Simultaneously, these immune cells secrete IL-8 when stimulated by *Mycobacterium tuberculosis*. In addition, the expression levels of CYP1B1, IMPA1, FXR2, ECE1, LYZ, BHLHE41 and MARCH3 were also significantly changed. CYP1B1 is a member of the CYP superfamily and crucial for endogenous metabolic pathways regulation and is expected to be a therapeutic target for the treatment of metabolic diseases [58]. Owing to involvement in key aspects of cell metabolism, CYP1B1 is used in the diagnosis and predictive diagnosis of various diseases. Based on importance and function of mRNA more than conventional translation, CYP1B1 may also serve as a universal cancer marker. The viral target Endothelin-converting enzyme 1 (ECE1), as an IFNα2a-stimulated protein was exclusively upregulated at the surface of CD4^+^ T cells, is a new type of cell type specific IFN stimulating factor [59]. BHLHE41 belongs to the basic helix‐loop‐helix family member e41. It is a negative regulatory factor that regulates the inhibition of circadian rhythm, cell differentiation and apoptosis [60, 61]. Membrane-associated RING-CH 3 (MARCH3) is a member of the MARCH ubiquitin ligase family. Several members of this family have been shown to ubiquitinate and down-regulate transmembrane proteins, such as IL-1RAcP [62]. Furthermore, research by Heng Lin et al. showed that MARCH3 is a key negative regulator of IL-1β triggering signal. Overexpressed MARCH3 inhibits IL-1β from activating the expression of NF-κB and inflammatory genes, while inadequate MARCH3 has the opposite effect [63].

In GO analysis, lncRNAs are mainly enriched in protein homodimerization activity, positive regulation of angiogenesis, interleukin-8 receptor binding, lithium ion binding, trabecular meshwork development, regulation of single stranded viral RNA replication via double stranded DNA intermediate, and endothelial cell–cell adhesion, etc. The results of differential gene enrichment analysis in KEGG pathway showed that most of the genes enriched involved pathways related to the activation of host immune responses to mycobacteria and their regulatory mechanisms. The recognition of *Mycobacterium paratuberculosis* by macrophages is mediated by host pathogen recognition receptors, including Toll-like receptors (TLRs) and NOD-like receptors (NLRs). TLRs are essential in the recognition of pathogen associated molecular patterns (PAMPs). TLR is a member of mammalian pattern recognition receptor, which is a key component of pathogen recognition mechanism of inflammatory response induced by a microorganism or microbial cell component. TLRs can not only mediate the up-regulation of interleukins, chemokines, costimulatory molecules, adhesion and pro-inflammatory/anti-inflammatory cytokines, but also guide phagocytes to process and present antigens, and induce their own expression [64]. The intracellular NOD-like receptor (NLR) family contains more than 20 members in mammals and is important in the recognition of intracellular ligands. It can be expressed in many cell types, including immune cells and epithelial cells, and thus playing an important role in host innate immune responses and immune homeostasis. Most NLRs are capable of acting as pattern-recognition receptors (PRRs), recognizing various ligands from microbial pathogens, host cells, and environmental sources and activating inflammatory responses. A study showed that NOD2 played an important role in regulating the inflammatory response and intracellular growth of *Mycobacterium tuberculosis* in human primary macrophages [65]. Additionally, the NF-κB pathway was significantly enriched. The NF-κB pathway helps control cell survival, differentiation and proliferation. Abnormal activation of NF-κB is associated with various diseases such as cancer, autoimmune disease, neurodegenerative disease and cardiovascular disease. Of these diseases, the role of NF-κB signaling in chronic inflammatory and autoimmune diseases, such as Crohn’s disease, ulcerative colitis, and rheumatoid arthritis, is well-defined and known. According to our results, cytokine-cytokine receptor interactions were also significantly enriched. Cytokines, primarily derived from mononuclear phagocytes and other antigen-presenting cells (APCs), are particularly effective in promoting cellular infiltration and resident tissue damage characteristic of inflammation. Cytokines are virtually involved in every aspect of immunity and inflammation, including innate immunity, antigen presentation, myeloid differentiation, cell recruitment and activation, and adhesion molecule expression. Depending on the source of cytokines, their functions are also different that mainly refer to mediating cytotoxic, humoral, cellular or allergic immunity. Moreover, IL-17 signaling pathway was also significantly enriched. The IL-17 cytokine family was named CTLA8 when it was discovered in 1993, and is secreted by T helper cell 17 (Th17). IL-17 plays a key role in protecting the host against extracellular pathogens. The IL-17 family signals via their correspondent receptors and activates downstream pathways that include NF-κB, MAPKs and C/EBPs to induce the expression of antimicrobial peptides, cytokines and chemokines.

In a word, by transcriptome sequencing, a large number of biological information and multiple cell signaling pathways in response to infection was obtained. Based on results of the study, it can provide a basis for comparing the differences of macrophages’ responses to different mycobacteria, and help to clarify the key cellular pathways involved in the early stage of infection. Moreover, screening gene expression information related to infection can also provide a theoretical basis for paratuberculosis diagnosis improvement.

Research on the regulatory relationship network and target gene function in our study is mainly based on lncRNA, analysis and prediction of lncRNA-mRNA transcriptome data. Among the possible modes of action, lncRNA may exert regulatory effect by inhibiting or stimulating gene expression. The potential role of lncRNA as a biomarker has been confirmed in *Mycobacterium tuberculosis* infection, and this may serve as a promising method in *Mycobacterium tuberculosis* infection. Furthermore, some lncRNAs are highly regulated by infection, but their functional role still needs to be studied and verified from the aspect of regulation mechanism. Whether the sequenced lncRNAs are specific and sensitive to MAP infection, and whether they can be potential biomarkers for the specific detection of infected animals remain to be verified in the future.

Briefly, in future studies, we will compare and analyze the differences in protein expression between MAP-infected and control groups, and perform association analysis of the three in combination with mRNA and lncRNA to improve the regulatory network of lncRNA. This will provide a new perspective for the pathogenic mechanism study of MAP or novel defense strategies of host cells.

## Material and methods

### Purification of bovine monocyte derived macrophages

Six healthy Holstein cows (3–6 years old) with no history of Johne’s disease were selected. Collect 200 mL of peripheral blood from the bovine jugular vein and place it in a collection container containing the anticoagulant sodium citrate. The collected blood was then subjected to density gradient centrifugation using lymphocyte separation medium. Peripheral blood mononuclear cells (PBMCs) in the buff layer were collected and resuspended in PBS containing 0.5% BSA and 2 mM EDTA (pH 7.2). Isolation of CD14^+^ cells was performed using CD14 MACS magnetic beads according to the manufacturers instructions. After magnetic bead sorting, the collected cells were cultured in RPMI 1640 medium containing 10% FBS (Invitrogen, Shanghai, China) and added with 1% non-essential amino acids and 50 ug/mL gentamicin (Sangon Biotech, Shanghai, China). The culture conditions were 37 ℃ and 5% CO_2_, and the culture density was 1 × 10^6^/well in 24-well cell culture plate. The culture medium was changed on the third day when the monocytes were cultured. At this time, the cell morphology was uniform as shown in Supplementary Fig. 2A. Through flow cytometry, the purity of monocytes after sorting can reach more than 95%, and the results are shown in Supplementary Fig. 2B. The cells were incubated overnight before being used for uptake and replication experiments and allowed to differentiate into the early adherent macrophage phenotype confirmed under the microscope (Supplementary Fig. 2C). When the cells differentiated to the 5th day, the monocyte-derived macrophages were infected by MAP in a 5: 1 infection plural number.

### Bacterial infection

The MAP-10 strain is preserved by the China Institute for Veterinary Drug Control and cultured in Middlebrook 7H9 supplemented with 2 mg/ml Mycobactin J and a 10% Middlebrook OADC enrichment (BD Biosciences, Shanghai, China). The absorbance of the bacterial suspension (OD 600 nm) was measured to evaluate the growth of the bacteria until the collected bacteria could be used for infection. After 8 weeks, bacteria were collected, dissolved in RPMI 1640 cell culture medium, washed and suspended again to prevent bacterial clumps. Finally, colony count was performed [66] and used to infect cells. For infection, one group was infected with MAP, at a multiplicity of infection of 5:1, the other group was not infected, and each group has three replicates. At 6 h post-infection, the cells were harvested, and the infected and non-infected control MDMs were lysed and stored at -80 ℃ until required for RNA extraction.

### RNA extraction, library preparation, sequencing and data analysis

Total RNA was extracted using Trizol reagent (Invitrogen, CA, USA) following the manufacturer’s procedure. The total RNA quantity and purity were analysis of Bioanalyzer 2100 and RNA 6000 Nano LabChip Kit (Agilent, CA, USA) with RIN number > 9.2. Approximately 1 ug of total RNA were used to prepare small RNA library according to protocol of TruSeq Small RNA Sample Prep Kits (Illumina, San Diego, USA).

Ribosomal RNA was removed and approximately 10 ug of total RNA representing the specific adipose type was used to deplete ribosomal RNA. After RNA was purified, the cleaved RNA fragments were reverse transcribed according to the scheme of mRNA-Seq sample preparation kit (Illumina, San Diego, USA) to create the final cDNA library. Then we performed double-terminal sequencing on an Illumina Hiseq 4000 (lc-bio, China) according to the scheme recommended by the supplier.

### Transcript assembly and lncRNA screening

After assembling reads using the latest transcript assembly software StringTie, known mRNAs and transcripts smaller than 200 bp were removed, and lncRNA prediction was performed on the remaining transcripts. The prediction software is CPC and CNCI. If these remaining transcripts had the potential to encode proteins, we classified them as novel mRNAs, which were then filtered after defining them as mRNAs. Through some filtering conditions, the lncRNA sequence is finally obtained.

After the final transcriptome was generated, estimate the expression levels of all transcripts, and then lncRNA was screened. Transcripts overlapping with known mRNAs and transcripts shorter than 200 bp were discarded first. Then, CPC and CNCI were used to predict the transcripts with coding potential. All transcripts with CPC score < -1 and CNCI score < 0 were removed. The other types of transcripts with code names I, J, O, U, X are lncRNAs.

Raw RNA-Seq fastq data for the mRNA and lncRNA discussed in this article have been deposited at NCBI and can be found under GEO series accession number GSE193595.

### Differential lncRNA expression analysis

For biological replicate results, the Ballgown package in R language was used to perform differential analysis of lncRNA genes assembled and quantified by StringTie. For the screening of differentially expressed genes, the differential lncRNAs were screened with log2 (Foldchange) > 1 (two-fold difference fold) and *P* value ≤ 0.05 as the threshold.

### lncRNA-mRNA network construction, target prediction and functional analysis

According to relevant studies, lncRNA and mRNA are different in many aspects, such as structural characteristics (length distribution, exon number, ORF length) and expression levels. Therefore, this section mainly conducts a systematic comparative analysis on the structural characteristics and expression levels of lncRNAs and mRNAs. To explore the function of lncRNAs, we predicted the cis-target genes of lncRNAs. LncRNAs may play a cis role acting on neighboring target genes. In this study, coding genes in 100,000 upstream and downstream were selected by perl script. Then, we showed functional analysis of the target genes for lncRNAs by using the scripts in house. Significance was expressed as a *P* value ≤ 0.05.

In this experiment, only cis-regulation predictions were made for the upstream and downstream 100 kbp mRNA and lncRNA. GO and KEGG enrichment analysis was performed on the targeted differential mRNA.

## Supplementary Information

**Additional file 1.**

**Additional file 2.**

## Data Availability

Raw sequencing files and associated metadata have been deposited in NCBI’s Sequence Read Archive (GSE193595).
