# Supplementary figures and images for: Analysis of long non-coding RNA expression profile of bovine monocyte-macrophage infected by Mycobacterium avium subsp. paratuberculosis
Source: BMC Genomics. 2022 Nov 24;23:768. doi: 10.1186/s12864-022-08997-5 (PMC9685057; doi:10.1186/s12864-022-08997-5)

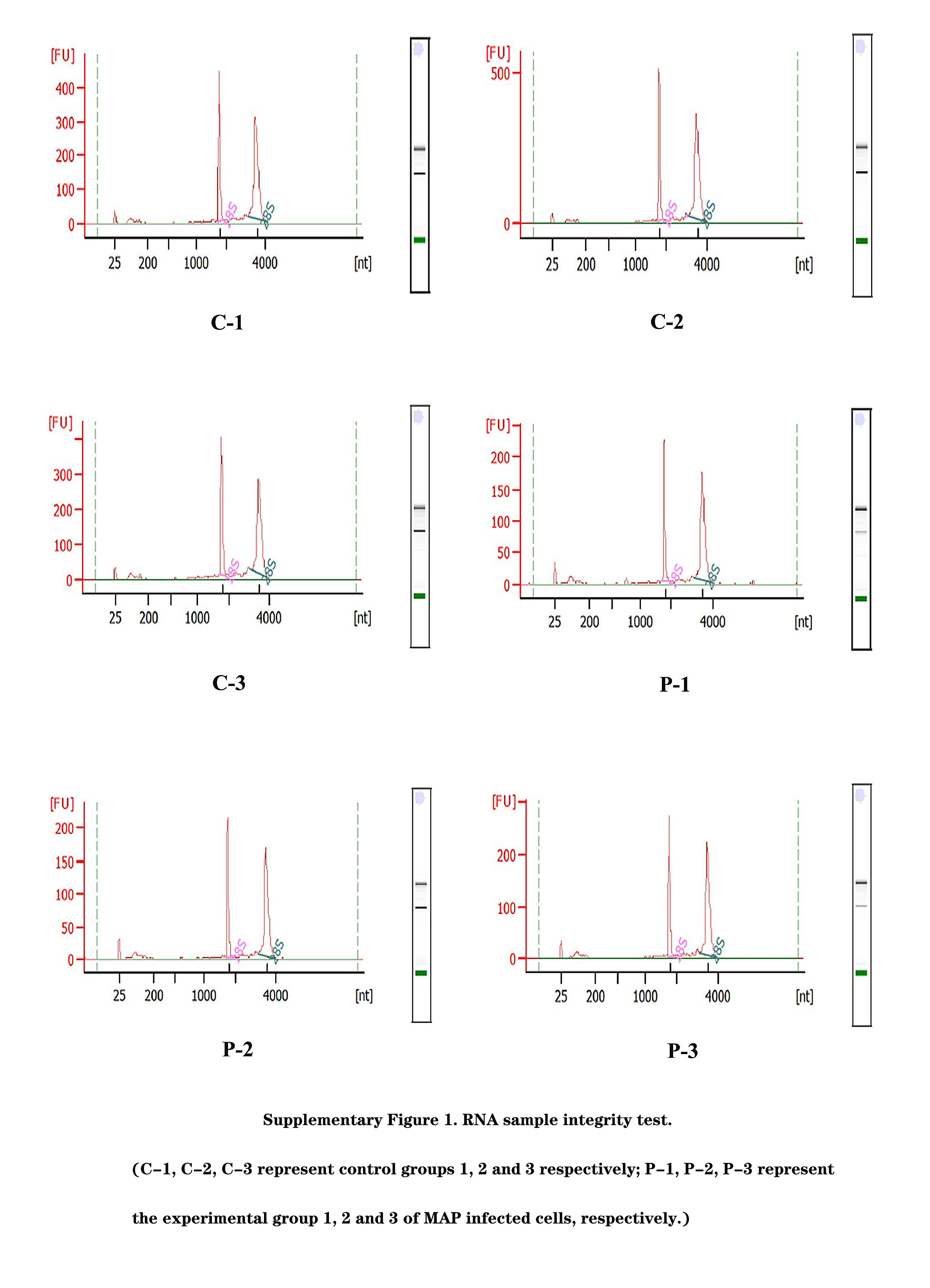

Supplement: Supplementary file 1 — Additional file 1. [file 12864_2022_8997_MOESM1_ESM.zip › Supplementary Figure 1-2 (22.11.02)/Supplementary Figure 1.tif]

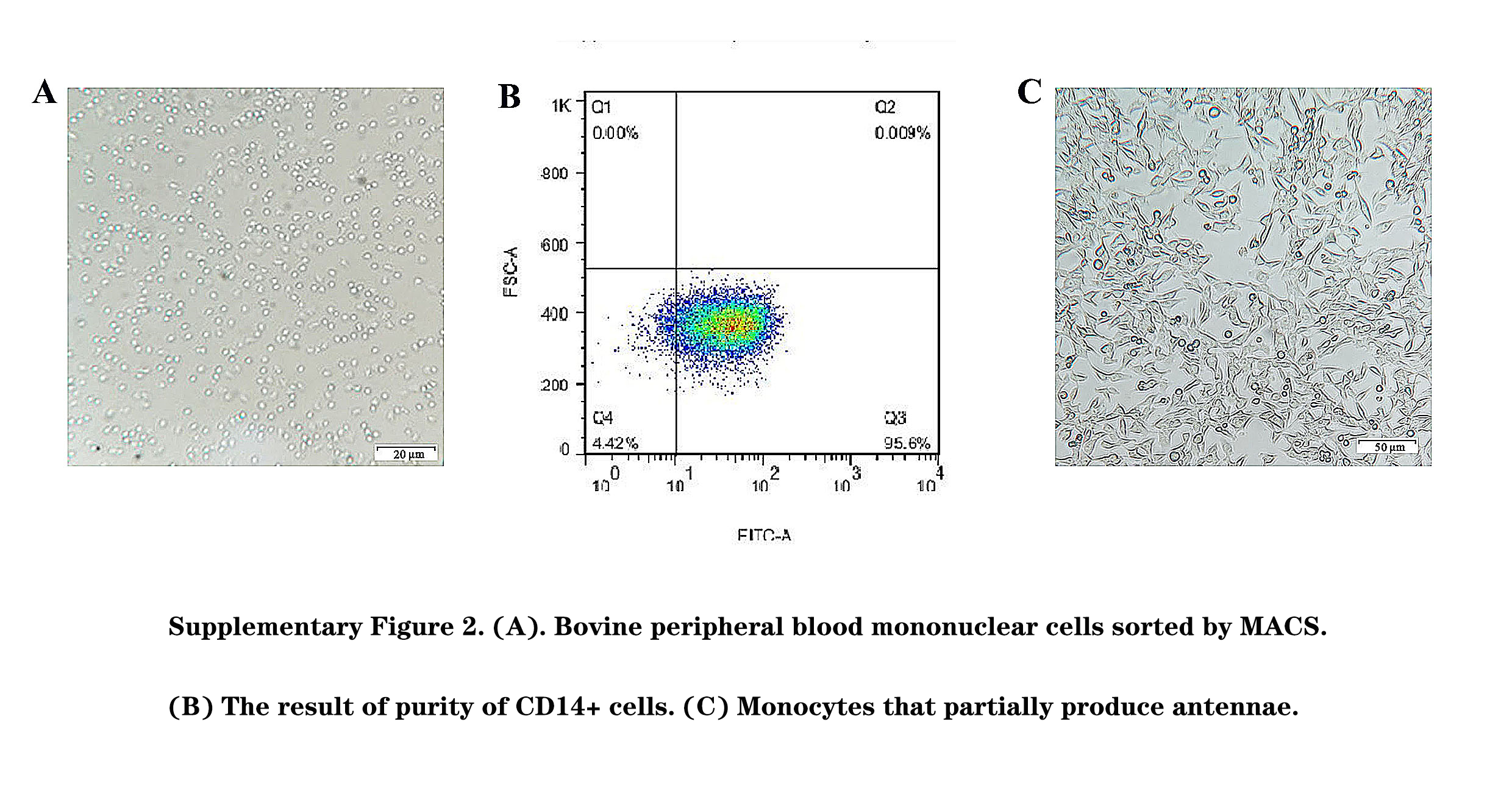

Supplement: Supplementary file 1 — Additional file 1. [file 12864_2022_8997_MOESM1_ESM.zip › Supplementary Figure 1-2 (22.11.02)/Supplementary Figure 2.tif]
